# Physical activity and sedentary time after lifestyle interventions at the Norwegian Healthy Life Centres

**DOI:** 10.1017/S1463423623000658

**Published:** 2024-01-08

**Authors:** Odin H. Gryte, Eivind Meland, Gro B. Samdal, Lars T. Fadnes, Jørn H. Vold, Thomas Mildestvedt

**Affiliations:** 1 Department of Global Public Health and Primary Care, University of Bergen, Bergen, Norway; 2 Faculty of Health, VID Specialized University, Norway; 3 Bergen Addiction Research, Department of Addiction Medicine, Haukeland University Hospital, Bergen, Norway; 4 Division of Psychiatry, Haukeland University Hospital, Bergen, Norway

**Keywords:** behavior change, lifestyle intervention, motivation, physical activity, sedentary time, self-rated health

## Abstract

**Aims:**

This study evaluates long-term changes in physical activity and its associations with various predictors after a behavior change program at the Norwegian Healthy Life Centers.

**Background:**

Physical activity is recommended and is part of public health strategies to prevent noncommunicable diseases.

**Methods:**

This longitudinal cohort, based on a controlled randomized trial, studies a population of 116 Healthy Life Center participants in South-Western Norway who wore SenseWear Armbands to measure time spent in moderate to vigorous physical activity and sedentary time based on metabolic equivalents. The measurements were obtained at baseline, immediately post-intervention, and 24 months after baseline. Linear mixed model analyses were performed to assess predictors for change in physical activity and sedentary time.

**Findings:**

High physical activity levels at baseline were maintained during the 24-month study period. Young, male participants with good self-rated health, utilizing local PA facilities were most active, and young participants utilizing local facilities were also less sedentary. The participants with higher levels of education were less active initially but caught up with the difference during follow-up. A high degree of controlled regulation, characterized by bad conscience and external pressure, predicted more sedentary behavior and a trend toward being less physically active. Autonomous motivation was associated with less time spent on sedentary behaviors. People with high self-efficacy for physical activity were more sedentary initially but showed a reduction in their sedentary behavior.

The study supports the importance of attending local training facilities and adopting motivation for behavioral change that is not based on guilt and external rewards. Interventions aimed at improving physical activity among people at risk for noncommunicable diseases benefit from habitual use of local training facilities, strengthening their self-perceived health and the development of internalized motivation. However, it has not been shown to mitigate social health disparities.

## Introduction

Noncommunicable diseases (NCDs) currently account for nearly two-thirds of deaths worldwide, and the emergence of chronic diseases as the predominant challenge to global health is undisputed (Bauer *et al*., 2014). The importance of physical activity (PA) in the pursuit of health and longevity is well documented and a systematic review and meta-analysis that included over 36,000 patients showed that higher levels of PA, at any intensity, and less time spent being sedentary, were associated with a reduced risk for premature mortality (Ekelund *et al*., 2019). The review reported a non-linear, dose-response pattern in middle-aged and older adults. It is also important to emphasize that sedentary behavior, such as sitting and prolonged time watching television, is associated with increased risk of chronic disease (Patterson *et al*., 2018).

In 2013, the World Health Organization (WHO) published a global action plan that encouraged governments to develop NCD strategies (WHO, 2013). Following this, The Norwegian Directorate of Health recommended the establishment of Healthy Life Centers (HLCs) in primary health care to support those who need help to change their health behavior (The Norwegian Directorate of Health, 2016). The target population is adults over 18 years old, who have an increased risk of developing NCDs, or who are already living with a chronic disease.

A recent Norwegian observational study of a 3-month intervention at 32 HLCs showed no increase in PA at 12-months’ follow-up, but the participants improved their health-related quality of life 15 months post-intervention. Those who increased their PA were more likely to improve their health-related quality of life (Blom *et al*., 2020).

A systematic review suggested that life events could heavily impact PA (Condello *et al*., 2017). For example, a transition to university, giving birth, or becoming a parent were probable causes of decreased PA for an unspecified period. There is also research supporting that the assumed connection between higher socioeconomic status and higher levels of PA is not as clear-cut (Stalsberg and Pedersen, 2018). Higher educated individuals may have more leisure time PA but fall short on PA throughout day-to-day life. The HLCs are part of a government policy to mitigate social health differences by recruiting participants from lower SES groups (The Norwegian Directorate of Health, 2016), but current HLC research has not found recruited participants to mainly be from lower SES groups (Samdal and Meland, 2022).

Self-determination theory (SDT) explains different types of motivational qualities (Ryan *et al*., 2008). Autonomous regulation derives from internal sources and personal satisfaction with a health behavior and increases the probability of initiation and maintenance of change. Controlled regulation is a result of bad conscience or social pressure to avoid punishment or to achieve an external reward. The importance of autonomous regulation has been confirmed in several exercise studies (Brunet and Sabiston, 2011, Teixeira *et al*., 2012). A meta-analysis of effective behavioral change techniques revealed that goal setting and self-monitoring of behavior were associated with dietary and PA improvements (Samdal *et al*., 2017). Interventions that used motivational interviewing or that were based on SDT were associated with success in both the short and the long term.

The Healthy Life Center Study is a six-month randomized controlled trial (RCT) with a 24-month follow-up period of participants attending behavioral change interventions at the Norwegian HLCs (Abildsnes *et al*., 2017).

The study aimed to explore the effects of the HLC interventions on physical activity, diet and eating behavior, body attitude, self-rated health, and well-being. Earlier studies revealed that participants were predominantly obese, physically active, female, and motivated for change. The trial reached socioeconomically disadvantaged groups, for example, low educational attainment and low incomes (Samdal *et al*., 2018). A low level of education did not explain drop-out, which was more likely among participants with chronic somatic disease and mental or musculoskeletal challenges. The intervention had no short-term effect on time spent physical active or sedentary compared with controls. Although less active people benefitted more from the intervention, the interventions were unable to counteract the widening of inequity across educational groups (Samdal *et al*., 2018). Many participants wanted help with high body weight. The trial revealed no weight difference between the intervention groups, and educational attainment did not differentiate change in weight or body attitude (Samdal *et al*., 2021). Higher levels of self-rated health (SRH) and autonomous motivation for change impacted weight loss. A beneficial body attitude was also predicted by life satisfaction and self-efficacy for PA. The participants who attended an additional healthy eating intervention, produced a modest, improvement in healthy eating after six months, but produced no effect on unhealthy eating compared with controls.

No effects of educational differences were revealed, and contrary to common beliefs, higher income predicted unhealthier eating as time passed (Samdal *et al*., 2021). The study revealed that healthy eating may be improved by an emphasis on developing positive self-concepts like better SRH, vitality, life satisfaction, and self-esteem. Together all these findings support a holistic approach to health behavior counseling, in line with the intentions put forward in the updated recommendations from Norwegian health authorities (The Norwegian Directorate of Health, 2016).

### Aims

This paper presents the 24-month follow-up of The Norwegian Healthy Life Study (Abildsnes *et al*., 2017). The study merged all participants from the RCT into one cohort. We aimed at examining changes in MVPA (moderate- to vigorous PA) and time being sedentary during the intervention and 24 months after baseline, and to evaluate the predictors of PA or being sedentary during the follow-up period. We specifically aimed at understanding facilitating and hampering predictors for long-term behavior change in a primary care setting.

## Methods

### Design

Participants included in the study had to be deemed eligible for the service provided by HLCs, aged 18 or above, and able to participate in a group intervention delivered in the Norwegian language. Participants with mental illness, learning disabilities, or those who only wanted a tobacco cessation intervention were excluded from the study.

### Setting

Twelve HLCs from municipalities in Southern and Western Norway were invited to participate in the study. Four of them declined the invitation, one due to other research commitments. The remaining eight municipalities represented 630,000 inhabitants from both urban and rural areas (6000–270,000 inhabitants). One of the HLCs served three municipalities, leaving six in total.

### The intervention

The intervention consisted of an individual 30–60-minute session where an HLC counselor provided information tailored to the participants’ abilities and needs and offered support for behavioral change based on a mutually agreed plan using motivational interviewing (Hettema *et al*., 2004). The PA intervention included exercises such as Nordic walking, light strength training, and competitive games. The different HLCs implemented their interventions depending on local policy, competence, and resources. The initial duration of the intervention was 12 weeks, with the possibility of prolongation up to 12 months.

### Study period and population

The six HLCs invited a total of 351 people to take part in the study from June 2014 to September 2015. The participants were either referred to the HLC by health professionals in the municipalities, or they came on their own initiative. Of the 351 people invited, 116 agreed to participate (33%). The participants were then divided into two intervention groups using a random number list aimed at providing an approximately equal distribution of participants per HLC. One of the groups started the interventions immediately, while the second group started their interventions six months later. We have combined the initial control- and intervention groups into one cohort for this study to examine the effects 24 months after intervention started. The main reason people gave for declining to participate in the study was the risk of a six-month delay in starting their intervention.

### Data collection

**Figure 1.**
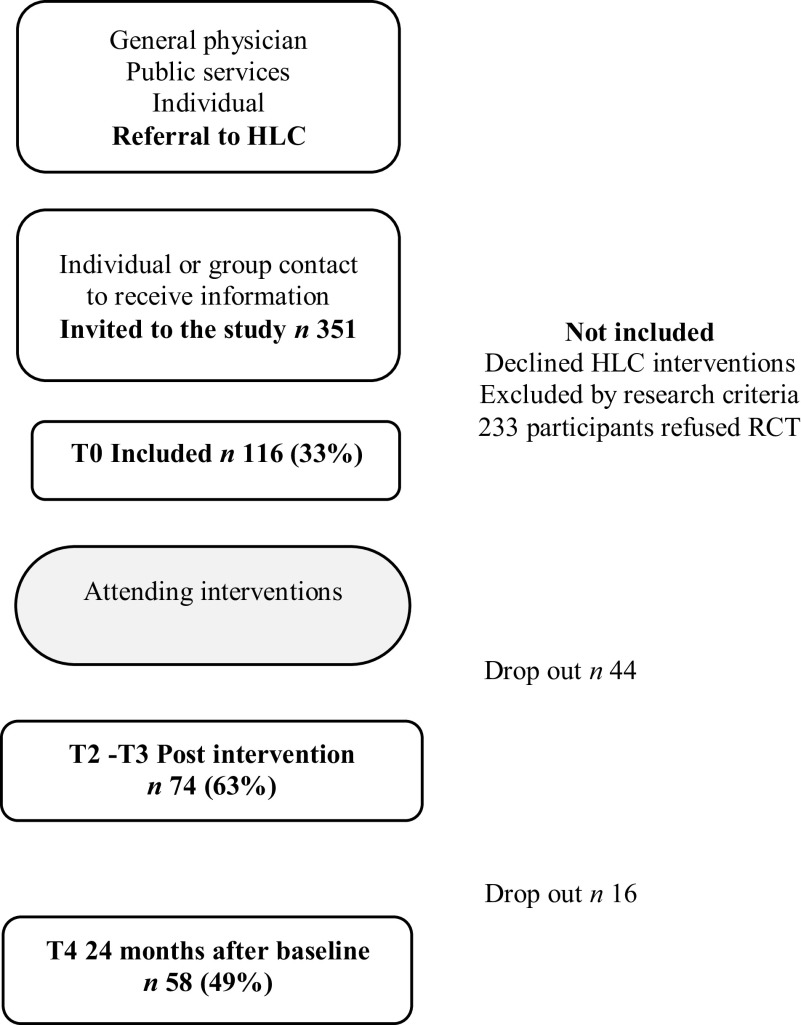
Flow chart of referral, uptake, drop out, and attendance in the Norwegian Healthy Life study. Abbreviation: HCL = Healthy Life Centre; number and percent of the remaining study participants

For both groups, we collected data at baseline before group allocation and the start of the intervention (T0), post-intervention (T2 and T3), and 24 months after baseline (T4). The last follow-up was 18 months post-intervention for the immediate intervention group, and 12 months post-intervention for the delayed intervention group (Figure 1) (Abildsnes *et al*., 2017).

We gathered data using an online survey management system (SurveyXact®; Rambøll Management Consulting, Oslo, Norway), and all surveys were completed at the HLCs. The survey was tested and approved by three participants from different HLCs.

#### Objective measurements of PA

Our primary outcome was MVPA, defined as ≥ 3 metabolic equivalents (METs). One MET is defined as the amount of oxygen consumed while at rest and equals 3.5 mL O_2_ /kg body weight × minutes (Jetté *et al*., 1990). We also defined sedentary behavior as < 1.5 METs. For objective measures, we used SenseWear Armband Mini (BodyMedia Inc., Pittsburgh, Pennsylvania, USA). Self-reported PA may be prone to discrepancies (Steene-Johannessen *et al*., 2016), although its validity has been confirmed compared with biological measures (Holen *et al*., 2012).

The HLCs instructed participants to wear the SenseWear Armband 24 hours per day for a seven-day week, except when showering or performing water-based activities. We registered the participants’ gender, height, weight, and age into the SenseWear Armband before each monitoring period. SenseWear Armband software (Version 7.0) transformed files of heat flow, acceleration, and other parameters into output measurements such as activity duration, steps, total energy expenditure, and on/off body time. From these data, we were able to compute the duration of time spent in MVPA as our primary outcome as well as sedentary time. However, we added two questions in the survey: ‘In general, for how long are you physically active each day?’; and ‘How hard do you exercise?’. The answers were categorized using a Likert score with five and four alternative responses respectively. The product of these two scores yielded a normally distributed composite score used in sensitivity analyses.

#### Predictors (explanatory variables)

The level of participant’s education was divided into three classes: Low (upper-secondary school or below), middle (upper-secondary school general studies), and high (university or university college). A questionnaire on self-efficacy for PA previously used in Norwegian studies was included (eight items) (Hansen *et al*., 2014). Questions were scored on Likert scales ranging from 0 to 6, where 0 indicates ‘not sure at all’ and 6 indicates ‘very sure’. Social support for PA (SSPA) provided by friends and family was identified by a six-item scale used in previous Norwegian surveys (Hansen *et al*., 2015, Sallis *et al*., 1987).

The variable ‘utilization of training facilities’ describes how often the participants use facilities such as gyms or sports stadiums for PA. SSPA and utilization of training facilities were both scored on a Likert scale 0–3, where 0 indicates ‘never or rarely’ and 3 indicates ‘often or very often’. SRH was measured by the question ‘how is your overall health at the moment?’ and categorized into groups: ‘bad/fairly bad’, ‘neither good nor bad’, and ‘good or very good’ (Vie *et al*., 2014).

Childhood experience of parental acceptance and respect is linked to emotional and behavioral adjustment (Rohner, 2004). We applied the statement; ‘I experienced respect and appreciation in my childhood’ (Likert scale 0–6) with response categories ranging from ‘0 – Strongly disagree’ to ‘6 – Strongly agree’. We included two motivational qualities from the Treatment Self-Regulation Questionnaire to measure the reasons why a person wants to change a bad habit or to continue a good one (Levesque *et al*., 2007): autonomous regulation (six items), and controlled regulation (six items), both rated on a Likert scale 0–6 from ‘never’ to ‘always’.

#### Dropout

We registered dropouts immediately post-intervention and at 24-months’ follow-up. At post-intervention, 44 of the 116 original participants had dropped out, with 74 (64%) participants remaining. Later, at the 24-month follow-up, 16 more participants had dropped out, with a total of 58 participants (49%) left in the study.

### Statistical analyses

We used the statistical analysis tool SPSS (IBM Statistical Package for Social Sciences version 26) for descriptive statistics and Stata/SE 17.0 (StataCorp, TX, USA) for the linear mixed model analyses (West and Welch, 2014, Hox *et al*., 2017). The threshold for statistical significance was set to *P* < 0.05 for all analyses unless otherwise stated. We defined time as the number of years from baseline. We accepted valid measurements from the SenseWear Armband if the participants wore the armband for at least 80% of a 24-hour day or 19 hours.

Missing values across predictor variables were handled by replacing the values by mean. Two participants (1.7%) had missing values in the variables. For scaled variables, we presented the mean value and standard deviation (SD) of the participants, whereas, for categorical variables, we described the number of participants (*n*) out of the 116 that belonged to a certain category and the percentage out of the total.

We performed linear mixed model analyses for the outcome variables MVPA or being sedentary, adjusted for the exposure variables at baseline; intervention group, sex, age, level of education, childhood experience of respect, controlled regulation, autonomous regulation, self-efficacy for PA, SSPA, utilization of training facilities, and SRH. All variables scored on Likert scales were scaled from 0 to 1 to simplify the interpretation for our mixed model analyses. The exposure variables were kept constant to the value held at baseline in the prediction of the levels and changes over time in the outcome variables. The exposure variables were set in interaction with time to calculate the time trends in the models.

We specified the linear mixed models as random intercept fixed slope regression models. The estimator was set to restricted maximum likelihood. The full information maximum likelihood ensured that all available measurements were used. Prediction graphs were created based on linear mixed model analyses to display changes in the outcome variables over time, adjusted for exposure variables. Mixed models, including all exposure variables and their interactions with time, are presented in supplementary files.

We performed a sensitivity analysis comparing MVPA with self-reported habitual PA (‘For how long are you physically active each day?’ and ‘How hard do you exercise?’ (Likert score 1−5, and 1−4, respectively) (Holen *et al*., 2012). We performed pairwise t-tests comparing baseline with post-intervention and baseline with 24 months of self-reports of physical activity.

### Ethical approval

We obtained informed consent from all participants before the interventions started. The experimental protocol was approved by the Regional Committee for Medical and Health Research Ethics, Norway (no xxxx).

## Results

Of the 116 recruited participants, 77% were women with a mean age (SD) of 48.5 years (13). Almost half (44%) of the participants had higher education from university or college. Only 16% rated their health as good or very good. At baseline, the participants had a mean (SD) MVPA in hours per day of 1.1 (1.0), and 79% achieved the Norwegian health authority’s recommendation of more than 150 minutes MVPA per week. The mean (SD) sedentary time was 19.4 (2.0) hours per day (Table 1).

**Table 1.** Descriptive baseline statistics of the 116 participants at baseline from the Norwegian Healthy Life Study RCT. Recruited between June 2014 and September 2015

Variable (scale)	*N* = 116
Women, *n (%)*	89 (77)
Age in years, *mean* (SD)	48.5 (13)
Highest education completed:	
Low: Upper-secondary school or below, *n (%)*	43 (37)
Middle: Upper-secondary school general studies, *n (%)*	22 (19)
High: University college and/or university, *n (%)*	51 (44)
Childhood experience of respect (0–6), mean (SD)	3.6 (1.8)
Utilization of training facilities (0–3), mean (SD)	1.5 (1.2)
Self-rated health:	
Bad or fairly bad, *n* (%)	65 (56)
Neither bad nor good health, *n (%)*	33 (28)
Good or very good health, *n (%)*	18 (16)
Self-efficacy for physical activity (0–6), mean (SD)	3.6 (1.6)
Autonomous regulation (0−6), mean (SD)	5.0 (0.9)
Controlled regulation (0−6), mean *(*SD)	3.0 (1.2)
Social support for physical activity (0−4), *mean* (SD)	2.2 (1.0)
Sedentary, in hours per day, mean (SD)	19.4 (2.0)
MVPA, in hours per day, mean (SD)	1.1 (1.0)
≥150 minutes of MVPA per week, *n (%)*	91 (79)

Abbreviations: SD = standard deviation; BMI = body mass index; NOK = Norwegian kroner; MET = metabolic equivalent; Sedentary <1,5 MET, MVPA = Moderate to vigorous physical activity (>3MET).

Not all participants wore their SenseWear armbands for one full week as instructed, although a majority did: 82 (71%) at baseline, 40 (59%) post-intervention, and 33 (59%) at the two-year follow-up. At all measurements, a maximum of 5% wore the armbands for three days or fewer, therefore, we included all participants with MVPA registrations in the analyses.

**Figure 2.**
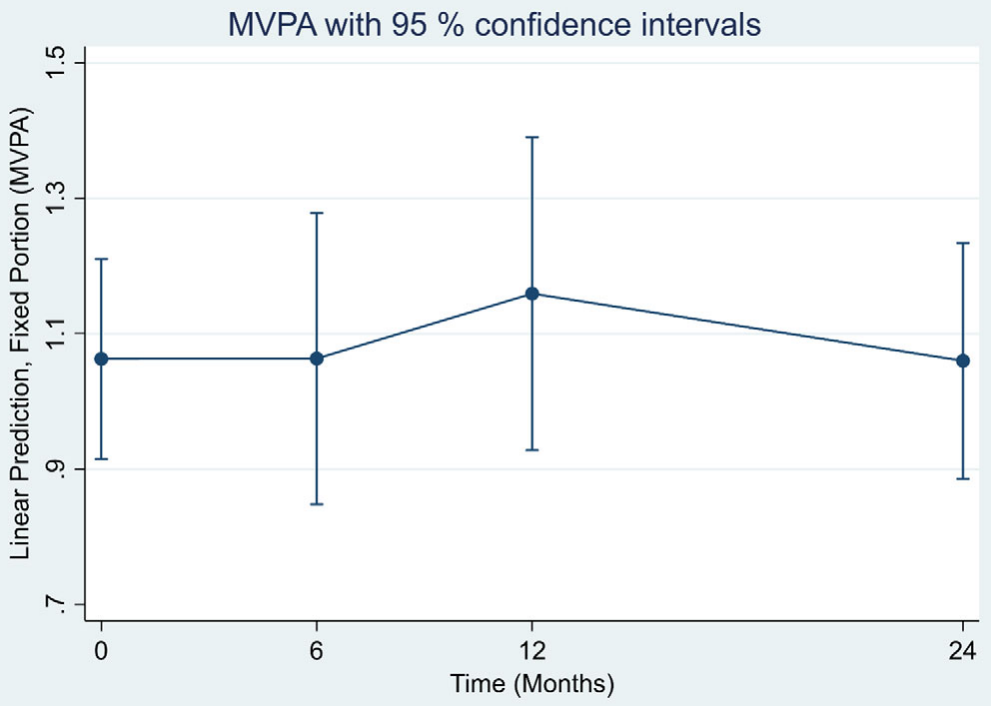
Linear prediction of MVPA (hours per day) at baseline and changes over time among 118 participants. Abbreviations: MVPA = moderate to vigorous physical activity

Figure 2 shows average time, in hours, spent in MVPA per day. High levels of PA at baseline were maintained across the 24 months. There was no significant change in MVPA.

**Figure 3.**
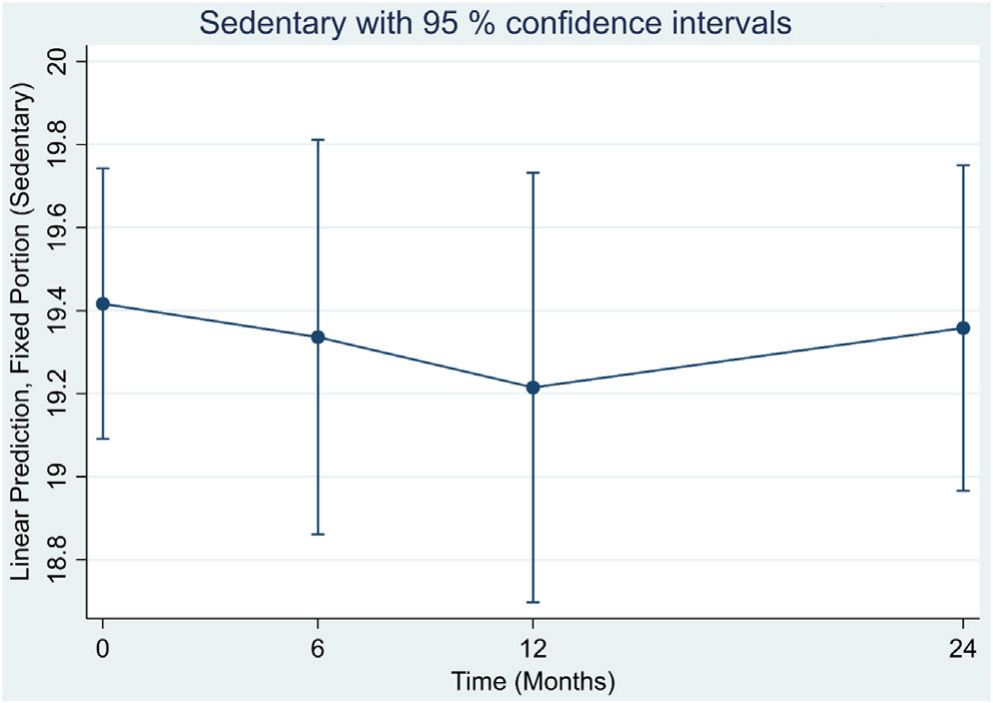
Linear prediction of sedentary time (hours per day) at baseline and changes over time among 118 participants

Figure 3 shows average time, in hours, sedentary time per day. Sedentary time did not increase over the span of 24 months.

Mixed model regression analysis of MVPA/week showed that at baseline, younger participants had higher MVPA than older participants (0.20, 95% confidence interval (CI): −0.32; −0.08) (Table 2). Females were less physically active (lower MVPA) than males (−0.74, 95% CI: −1.10; −0.38), and participants with high educational attainment had lower MVPA compared to those with low educational attainment (−0.39, 95% CI: −0.74; −0.05). Participants who scored high in controlled motivation were less physically active than those with low controlled motivation (−0.89, 95% CI: −1.76; −0.02). Using training facilities previously was associated with higher MVPA (0.59, 95% CI: 0.15; 1.02). People with good SRH were more physically active than their peers with poor subjective health (0.49, (95%CI 0.08; 0.90)).

**Table 2.** Mixed model regression of mean moderate to vigorous physical activity summary (MVPA/week) and association to motivation and other factors

MVPA	Baseline Coefficient (95%CI)	Time trend (per year) Coefficient (95%CI)
Intervention (versus control)	−0.01 (−031; 0.30)	0.01 (−0.14; 0.14)
Female (versus male)	−0.74 (−1.10; −0.38)	−0.01 (−0.17; 0.17)
Age (per 10-year increase)	−0.20 (−0.32; −0.08)	0.02 (−0.04; 0.08)
Low education	0 (reference)	0 (reference)
Middle education	−0.35 (−0.79; 0.09)	0.18 (−0.02; 0.38)
High education	−0.39 (−0.74; −0.05)	0.20 (0.03; 0.37)
Respect in childhood	0.18 (−0.35; 0.70)	0.19 (−0.05; 0.42)
Controlled regulation	−0.89 (−1.76; −0.02)	0.34 (−0.07; 0.75)
Utilizing PA facilities	0.59 (0.15; 1.02)	−0.10 (−0.30; 0.10)
Autonomous regulation	0.63 (−0.47; 1.73)	−0.07 (−0.62; 0.48)
SSPA	−0.40 (−1.12; 0.32)	−0.12 (−0.48; 0.24)
Self-efficacy for PA	−0.12 (−0.75; 0.50)	0.01 (−0.29; 0.30)
Self-rated health:		
Poor	0 (reference)	0 (reference)
Neither good nor bad	0.18 (−0.23; 0.59)	0.04 (−0.15; 0.22)
Good	0.49 (0.08; 0.90)	−0.06 (−0.25; 0.12)
Constant/time	2.40 (1.30; 3.50)	−0.32 (−0.86; 0.22)

Abbreviations: PA = physical activity; MVPA = hours per day spent moderately to vigorously physically active; SSPA = social support for physical activity. Education levels: Low (upper-secondary or below), middle (upper-secondary school general studies), and high (university college and/or university).

The gender difference observed at baseline was sustained over the 24 months. Those with higher educational attainment caught up with their less educated peers over time (0.20, 95% CI: 0.03; 0.37). Participants with higher controlled motivation at baseline tended to improve their MVPA over time (0.34, 95% CI: −0.07; 0.75). Participants utilizing training facilities and those with good subjective health maintained their MVPA.

The mixed model regression analysis of sedentary time showed that older and highly educated participants spent more time being sedentary than their peers at baseline (Table 3), with no substantial sex differences. Those with a high degree of controlled motivation were also more sedentary (1.98, 95% CI: 0.06, 3.90). However, those with a high degree of autonomous motivation tended to be less sedentary (−2.17, 95% CI: −4.59, 0.26). Further, participants who had used training facilities previously were less sedentary (−1.31, 95% CI: −2.27, −0.35). People with good SRH and high self-efficacy for PA tended to be less sedentary than their peers.

**Table 3.** Mixed model regression of mean hours/day of inactivity (being sedentary) and association to motivation and other factors

Sedentary	Baseline Coefficient (95%CI)	Time trend (per year) Coefficient (95%CI)
Intervention (vs. control)	−0.07 (−0.73; 0.60)	0.05 (−0.28; 0.38)
Female (vs. male)	−0.08 (−0.87; 0.71)	−0.07 (−0.47; 0.32)
Age (per 10-year increase)	0.39 (0.13; 0.66)	0.04 (−0.11; 0.19)
Low education	0 (reference)	0 (reference)
Middle education	0.53 (−0.44; 1.50)	−0.16 (−0.64; 0.32)
High education	1.02 (0.25; 1.78)	−0.51 (−0.91; −0.11)
Childhood experience of respect	−0.93 (−2.08; 0.23)	0.04 (−0.52; 0.60)
Controlled regulation	1.98 (0.06; 3.90)	−0.56 (−1.53; 0.42)
Utilizing PA facilities	−1.31 (−2.27; −0.35)	0.18 (−0.29; 0.66)
Autonomous regulation	−2.17 (−4.59; 0.26)	0.38 (−0.94; 1.69)
SSPA	−0.34 (−1.93; 1.26)	0.60 (−0.28; 1.47)
Self-efficacy for PA	1.19 (−0.19; 2.57)	−0.35 (−1.05; 0.35)
Self-rated health:		
Poor	0 (reference)	0 (reference)
Neither good nor bad	−0.60 (−1.50; 0.29)	0.04 (−0.40; 0.48)
Good	−0.82 (−1.73; 0.08)	−0.09 (−0.53; 0.34)
Constant/time	19.03 (16.60; 21.46)	−0.20 (−1.48; 1.08)

Abbreviations: PA = physical activity; SSPA = social support for physical activity; Education levels = Low (upper-secondary or below), middle (upper-secondary school general studies), and high (university college and/or university).

Participants with higher education reduced their sedentary time significantly over time (−0.51, 95% CI: −0.91; −0.11). However, no other exposure variables were associated with changes in sedentary over time.

The sensitivity analysis revealed that self-reported habitual PA was highly significantly correlated with measured MVPA (*r* = 0.48, *P* < 0.001). Pair-wise T-tests showed that self-reported habitual PA increased significantly from baseline to post-intervention (*t* = −2.9, *P* 0.005), with a non-significant change 4 months after baseline (*t* = −1.2, *P* 0.25). We performed a drop-out analysis with independent t-tests or cross-table Chi-square tests to examine if drop-out was associated with factors at baseline (not shown in tables). Participants with lower levels of MVPA dropped out slightly more frequently than others (*t* = 2.12, *P* 0.04). Dropouts did not differ significantly from completers concerning sedentary time, age, BMI, gender, education, marital status, the presence of mental and psychological health complaints, self-rated health, or self-esteem.

## Discussion

### Main results

The HLC participants were able to maintain a high level of MVPA over 24 months. No substantial changes in MVPA or sedentary behavior were revealed in the study period. Participants with higher educational attainment started with low PA levels but caught up with their peers with lower educational attainment. Controlled motivation hampered PA and promoted sedentary behavior. However, autonomous regulation had no influence on PA. Higher levels of SRH were associated with improved PA, and this was sustained during the study period. Our participants were highly motivated for change and had already attained high levels of MVPA. People, in general, are more likely to take part in an intervention program when their motivation is high and the barriers to change are low (Rothman A, 2011).

Different lifestyle intervention programs have struggled to demonstrate long-term maintenance of PA. An extensive meta-analysis evaluating short and long-term effects of similar interventions showed substantial short-term but diminishing long-term effects (Samdal *et al*., 2017). A recent Norwegian study from 32 different HLCs showed no significant increase in PA at a 15-month follow-up (Blom *et al*., 2020). The literature shows conflicting results concerning effects of lifestyle interventions (Orrow *et al*., 2012, Pavey *et al*., 2011).

Associations between PA and education levels are inconsistent (Stalsberg and Pedersen, 2018). A common belief is that highly educated people are more physically active than people of lower socioeconomic status. Our study revealed the opposite, although the socioeconomic differences were mitigated over time.

In line with multiple studies, controlled motivation hampers initiation and maintenance of changes after behavior change interventions (Ryan *et al*., 2008). In contrast to other findings, autonomous motivation had no significant impact on PA levels in the present study. However, a ceiling effect of autonomous regulation seems a plausible explanation for this. A recent Norwegian qualitative study confirmed the importance of internal motivation in the pursuit of behavioral goals (Sevild *et al*., 2020). In line with earlier research (Breidablik *et al*., 2009), we confirmed that good SRH was associated with higher levels of PA at baseline and maintenance of PA during the study period. Participants in a similar study reported high levels of psychological distress. The results emphasize the importance of addressing these measures in behavioral change interventions (Sevild *et al*., 2020). This study also revealed impaired SRH.

We may question the validity of the PA measurements. In our study population, 79% already met the Norwegian health authority’s recommended 150 minutes of MVPA per week before the interventions started. A representative population study of 3000 individuals showed that only one in five met these recommendations (Hansen *et al*., 2012). Self-monitoring of behavior seems an effective technique in counseling overweight and obese adults in meta-analytic studies (Hannan *et al*., 2019, Samdal *et al*., 2017). Considering this, we may suspect that the high levels of MVPA were due to wearing the SenseWear Armband. However, the sensitivity analyses revealed that objectively measured MVPA and self-reported habitual PA are highly correlated. Further, the pair-wise t-tests confirmed the results of the MVPA measurements at long-term follow-up.

Previous results from the Norwegian Healthy Life study together with this study support a holistic approach to health behavior counseling, in line with the intentions put forward in the updated recommendations from Norwegian health authorities (The Norwegian Directorate of Health, 2016). Socioeconomic level is related to health and health behaviors at a population level. Based on this and the previous HLCs results, we may conclude that the interventions do not seem to mitigate social inequalities in health. On the other hand, we have no indications from the HLC research that health disparities are reinforced. However, population strategies with systemic efforts in communities, workplaces, schools, and leisure time activities are called for to improve population health and mitigate health disparities (Sniehotta *et al*., 2017).

### Strengths and limitations

The strengths of this study are that we managed to recruit and follow up a heterogenic group from both urban and rural communities, where participants had different socioeconomic backgrounds. We also collected data 24 months after baseline.

Of the 351 participants of Norwegian HLCs invited to the study, only 116 of them (33%) accepted the invitation. This made our study prone to selection bias, with restricted external validity. The participants who declined our invitation mostly did so due to the risk of being assigned to the control group (delayed intervention), where they were reluctant to wait six months to start their interventions.

The internal validity of our results could be threatened by the dropout rate accounting for 52% of the initial 116 participants. We examined if drop-out was associated with factors at baseline. A slight but statistically significant difference in MVPA was revealed. Otherwise, drop-outs were similar to completers. Bias from dropout depends on the analysis method, the type of data that is missing, and the effect that is estimated (Bell *et al*., 2013). However, replacing missing data with mean values in our analyses slightly reduces the risk of this threat. Still, we acknowledge that dropout affects the statistical power of the study and makes it prone to type two errors.

Further, devices such as the SenseWear Armband have been criticized for not being ideal for measuring energy expenditure in high-intensity PA in an observational study of standardized exercises, and in a practical guide for measuring PA (Santos-Lozano *et al*., 2017, Sylvia *et al*., 2014). These armbands would more accurately measure low to moderate-intensity PA and sedentary behavior. However, there are exercise-specific algorithms used to try to correct the potential error in measurements, but these algorithms have limitations if the type of exercise and its duration are unknown (Sylvia *et al*., 2014). Since our primary outcome was MVPA and the armbands seem to work well for moderate PA, we can assume that the total time spent ≥ 3 METs would remain reliable. Even if we might not have an accurate measurement of vigorous PA, we were only interested in the total combined time of moderate and vigorous PA.

The duration of the HLCs’ intervention was 12 weeks with the possibility of prolongation for each of the two groups. It is unclear if PA interventions are dose-response dependent. A scoping review in communities in the Nordic countries found no additional increase in PA or fitness from long-term (≥12 months) interventions compared with interventions of less duration (Haverinen *et al*., 2022, Samdal *et al*., 2017). In the present study, 83% of participants attended the HLC interventions for more than three months, therefore, it does not seem likely that additional intervention duration should affect the outcome.

Although a majority wore the SenseWear armbands for a full week as intended, the representativeness of the measurements might be reduced as some of the participants had fewer days with activity registrations. However, only a maximum of 5% had three or fewer registration days at any time point. We, therefore, chose to include all participants with registrations to maintain statistical power, being aware that the American Heart Association recommends measuring for seven days to obtain a habitual PA profile (Strath *et al*., 2013).

## Conclusion

Interventions aimed at improving physical activity among people at risk for noncommunicable diseases benefit from habitual use of local training facilities, strengthening their self-perceived health, and avoiding controlled and externalized motivation. We have no indications that health disparities are mitigated. Together with other results from the Norwegian Healthy Life study, this study also supports a holistic approach to health behavior counseling.
